# Nurses' Tribute Fund

**Published:** 1918-05-11

**Authors:** 


### NURSES' TRIBUTE FUND.

### Influential Meeting at Leicester.

A largely attended meeting was held at the Leicester Royal Infirmary on April 25, arranged by the institution in support of the National Tribute Fund for Nurses and the College of Nursing. Mr. T. Fielding Johnson, jun., High Sheriff of Leicestershire and Chairman of Governors, presided over an influential and largely attended assembly.

**Sir ARTHUR STANLEY,** G.B.E., M.P., who had a very cordial reception, interestingly reviewed the circumstances which led to the inception of the College of Nursing. The nursing profession had long required something in the nature of a standardisation of certificates. There were training-schools all over the country, differing very much in their training, and differing very much in the standard of efficiency of the nurses they sent out with certificates. In a training-school like that of the Leicester Infirmary their certificate would be accepted all the world over, but there were others where the authorities had little knowledge of what the value of a certificate was, and their certificate was frequently given to those without the proper qualifications. They found that nurses generally were very willing to take up the idea of a College, though there had been a great deal of misunderstanding about their aims and objects. For some reason the idea arose that it was what they called an association of employers and governors of hospitals who were trying to "enslave" the nursing profession even more than it was enslaved already. For himself he had never seen any signs of slavery about nurses. They had something like 2,500 members at this time last year; when the last report was published they had 7,000 and he believed at the present moment the number was over 9,000. He saw no reason why a year hence they should not have 20,000 members, and a demand by such a body for State recognition would be very hard to resist. With regard to the development of the scheme, while it was proper that the head centre of the College should be London, it was felt that large towns like Leicester should be formed into centres, and he congratulated Leicester on the fact that it was one of the pioneers in that direction, having already formed its own centre.

**The DUKE OF RUTLAND** said that never in the whole history of England had women loomed so largely in the public eye as at the present moment, thanks entirely to the splendid work they were doing in all directions, and not least in the nursing of the wounded, for the successful prosecution of the war. He believed that Leicester would in no way prove behindhand in its support of this great movement.

**Col. BOND** said they could not speak in too glowing terms of what English nurses were doing in France. They must not forget, however, the work which the nurses at home had been doing, not only the military nurses in their great military hospitals in England, but also the work which nurses had been doing among the civilian population—the District and Poor-Law nurses, who had been carrying on a great work with depleted ranks. He imagined really that the inwardness of that meeting was that the British Women's Hospital Committee were suggesting to the Empire that the people could not show their gratitude to the nurses in a better way than by subscribing to the College of Nursing, which was going to be the great educational centre for promoting efficiency, for promoting a standard of nursing, and for binding the nursing profession together. He did not think they could have selected a better time than the present for the carrying out of that great scheme.

A number of cheques were handed in. Mr. Martin Harvey gave a dramatic interpretation of "A Hymn of Love for England."

